# Childhood trauma and differential response to long-term psychoanalytic versus cognitive–behavioural therapy for chronic depression in adults

**DOI:** 10.1192/bjp.2024.112

**Published:** 2024-10

**Authors:** Lina Krakau, Mareike Ernst, Martin Hautzinger, Manfred E. Beutel, Marianne Leuzinger-Bohleber

**Affiliations:** Department of Psychosomatic Medicine and Psychotherapy, University Medical Center of the Johannes Gutenberg-University Mainz, Mainz, Germany; Department of Clinical Psychology, Psychotherapy and Psychoanalysis, Institute of Psychology, University of Klagenfurt, Klagenfurt am Woerthersee, Austria; Department of Psychology, Section Clinical Psychology and Psychotherapy, Eberhard Karls University, Tübingen, Germany

**Keywords:** Depressive disorders, trauma and stressor-related disorders, psychotherapy, cognitive–behavioural therapy, psychodynamic therapy

## Abstract

**Background:**

Childhood trauma is a major risk factor for chronic depression. It has been suggested that adults with chronic depression who have experienced childhood trauma may require long-term treatment owing to a breakdown of basic trust and related difficulties in developing a productive therapeutic relationship.

**Aims:**

As empirical studies have been preliminary and scarce, we studied the effects of psychoanalytic therapy (PAT) versus cognitive–behavioural therapy (CBT) for chronic depression in adults with a history of childhood trauma. In this subgroup, we expected a greater symptom reduction in PAT compared with CBT.

**Method:**

In a large trial of long-term psychotherapies for chronic depression (LAC-Study; Clinical Trial Register ISRCTN91956346), 210 adults received open-ended CBT or PAT in an out-patient setting and were examined yearly over 5 years on the Beck Depression Inventory – II (BDI-II). Based on a linear mixed model approach, we tested participant-reported childhood trauma based on the Childhood Trauma Questionnaire (CTQ) as a predictor and moderator of treatment outcome. CTQ subscales were examined exploratively.

**Results:**

Depressive symptoms decreased over time (*b* = −4.55, s.e. = 0.90, 95% CI −6.32 to −2.81, *T* = −5.08; *P* < 0.001). A significant three-way interaction between childhood trauma, time and therapy group (*b* = −0.05, s.e. = 0.02, 95% CI −0.09 to −0.01, *T* = −2.42; *P* = 0.016) indicated that participants with childhood trauma profited especially well from PATs.

**Conclusions:**

Our results indicate differential benefits from PAT compared with CBT among adults with chronic depression and a history of childhood trauma. The results have important implications for differential indication and policy.

People with a history of childhood trauma are at higher risk for recurring and persistent episodes of depression (chronic depression). Meta-analyses disagree on whether depressed individuals with a history of childhood trauma show a less favourable response following first-line depression treatment^1,2^ or not.^3^ It is suggested that although this patient group does profit from active treatment, they suffer from more residual symptoms and relapse risks.^4,5^ In representative community surveys approximately 8–12% of respondents report having experienced multiple forms of adversity,^6,7^ with each occurrence elevating the likelihood of subsequent incidents.^8^ In most cases, they suffered these acts of harm in the immediate caregiving setting, for example at the hands of their parents. Specifically, Hurren et al^9^ found that 78.5% of perpetrators of child abuse victimise their biological children. Step-parents and in-home caregivers constitute other important perpetrator groups.^9,10^

Traumatic experiences are associated with extreme feelings of helplessness and hopelessness.^11^ In the context of caregiving, these profoundly affect early relationship experiences and secure attachment, which form the base of future perceptions, emotions and behaviour in relationships and beyond.^12^ Basic trust in significant others, and the development of self-agency, may be severely compromised.^13,14^ Other people, for example therapists, are often perceived as critical, rejecting and potentially dangerous.^15^ The inadequate early relationship dynamic may be repeated inside and outside the therapy room,^16^ making it more difficult to form a helpful therapeutic alliance.^17^ The empirical literature has been inconclusive about differential benefits from different psychotherapy modalities in this patient group.^18–23^ Although previous research mostly focused on short-term psychotherapies, the effectiveness of long-term treatments for people with chronic depression and childhood trauma has been understudied.^24,25^ One of the few studies including psychodynamic therapies found that patient reports of greater family unhappiness and parental problems predicted a relatively stronger response to psychodynamic short- and long-term treatments compared with solution-focused therapy, which is more problem- and future-oriented and less focused on past experiences and their connections to present interpersonal relationships.^21^

## The current study

Data are drawn from the Outcomes of Long-term Psychotherapies of Chronically Depressed Patients (LAC) study^25–27^ comparing PAT and long-term CBT for chronic depression over a period of 5 years following treatment start. Patients could choose to be randomised or allocated to their preferred treatment option. The main outcomes at the 5-year follow-up were reported in Beutel et al.^28^ No differences in symptom reduction were found between CBT and PAT or allocation type, albeit with larger therapy doses in PAT. Greater structural change was achieved in PAT from the third year onward. The current analysis aimed to expand on the differential indication for people with chronic depression and determine the differential benefits of CBT and PAT for those with or without a history of childhood trauma. As a prominent risk factor complicating the course of depression, childhood trauma represents important diagnostic information that may help to improve treatment personalisation and clinical management. The study manuals clearly differ in their focus in the treatment of depression. PAT has a stronger treatment focus on the exploration of life-story narratives of past and interpersonal experiences and on working on their repercussions in the therapeutic relationship. It attempts to understand the symptom presentation within the context of disrupted developmental processes, specifically attempting to deal with unconscious fantasies and conflicts resulting from this disruption, which are then worked through in the ‘here and now’ of the therapeutic relationship.^15,29^ CBT focuses on cognitive dysfunctions, irrational thoughts and belief systems resulting from adverse childhood experiences and teaches skills to cope with symptoms.^30^ The manualised treatments were reliably discriminated by the Comparative Psychotherapy Process Scale (CPPS).^25,31^ Given PAT's specific treatment focus and higher amount of sessions, we hypothesised that people with chronic depression reporting a history of childhood trauma benefit more from psychoanalytic compared with cognitive–behavioural treatment.

## Method

### Study design and participants

For an extensive overview of the study design, we refer to previous publications.^25–28^ The study included *N* = 252 participants with chronic depression. Participants had to be between 21 and 60 years old, score ≥17 on the Beck Depression Inventory – II (BDI-II),^32^ receive a score ≥9 points on the Quick Inventory of Depressive Symptomatology Clinician Rating (QIDS-C)^33^ and meet the diagnostic criteria for major depression or dysthymia. If participants used antidepressant medication, they had to have been on a stable dosage for ≥4 weeks. Exclusion criteria comprised psychotic illness, substance misuse, dementia, borderline, schizoid, paranoid and antisocial personality disorder, acute and/or chronic physical illness and acute suicidality. Written informed consent was obtained from all participants. They were informed about the trial treatments (long-term CBT or PAT) and could choose to be randomised to CBT or PAT or to select their preferred treatment. The study's four-arm design included randomised or preferred CBT or PAT respectively. Participants were randomised by an independent statistics centre, generating separate random allocation sequences for the four study sites. The study sites were university-affiliated research institutions with out-patient clinics (university hospitals in Frankfurt, Mainz, Berlin and Hamburg, Germany) that coordinated diagnostics, training workshops and patient referral. Session frequency and treatment duration were not predetermined. For sample size calculation, see Beutel et al^26^ and Leuzinger-Bohleber et al.^25^ The study was registered (Clinical Trial Register ISRCTN91956346). The authors assert that all procedures contributing to this work comply with the ethical standards of the relevant national and institutional committees on human experimentation and with the Helsinki Declaration of 1975, as revised in 2008. All procedures involving human patients were approved by the Ethics Committee of the Physician Board of Rhineland-Palatinate, Mainz, Germany (Ref: 837.124.075659).

### Interventions

Psychoanalytic therapy (PAT) for depression has been well described.^15^ Study therapists were trained in the empirically validated manual for the treatment of chronic depression.^29^ The study manual integrates the treatment of people with chronic depression with research on embodied memories and early trauma.^15^ It is recognised that childhood trauma overwhelms the individual's capacity to maintain a minimal sense of safety and destroys trust in the availability of reliable and empathic others as well as one's own self-agency. Procedural, embodied memories developed before full maturation of memory structures are understood to unconsciously determine the individual's emotions, thoughts and actions in relationships in the present. Unprocessed, unconscious conflicts of the past can be repeated and understood in the transference and successively transferred into a healing process.

Cognitive–behavioural therapy (CBT) for depression is based on the work of Beck, Lewinsohn and others, and is integrated into a widely used and well-accepted treatment protocol in Germany.^30^ CBT therapists used five modules (problem analysis, goals, psychoeducation and rationale for treatment; behavioural activation and increasing pleasant activities; cognitive interventions to restructure basic assumptions and schemata; social skill training, problem-solving and stress management; maintenance and relapse prevention). From a cognitive–behavioural view, threat-related information processing, heightened emotional reactivity and disrupted reward processing have been formulated as treatment targets in the context of chronic depression and childhood trauma.^34^

Compared with CBT, PAT scored higher on items characteristic of psychodynamic interpersonal therapy (e.g. feelings and perceptions linked to past experiences; focus on the patient–therapist relationship) with moderate effect sizes (0.35–0.74), whereas CBT was rated higher (0.30–0.84) on cognitive–behavioural items (e.g. focus on irrational belief systems; teaching specific techniques). The interrater reliability was high (intraclass correlation coefficient >0.85).^25^ A difference in total session numbers between CBT and PAT may be considered as an intrinsic feature of the treatment approaches^35^ regulated by German guidelines, with CBT comprising up to 80 and PAT up to 300 sessions covered by insurance.^36^

### Instruments

Depressive symptoms were measured yearly over the course of 5 years using the Beck Depression Inventory – II (BDI-II).^32^ The instrument incorporates 21 statements rating the severity of depressive symptoms during the past 2 weeks on a 4-point Likert scale (0–3). Higher values denote higher symptom load. The German language version of the BDI-II has shown good reliability, sensitivity to change and validity.^37^ Good internal consistency was replicated in our sample (ω_baseline_ = 0.82).

Childhood trauma was measured with the Childhood Trauma Questionnaire (CTQ).^38,39^ A global scale and six subscales assess self-rated emotional abuse and neglect, physical abuse and neglect, sexual abuse, and family inconsistency, specific to the German version^40^. Family inconsistency refers to a subjective appraisal of instability, unsafety and unpredictability in the home and the relationship with the main family members more generally (e.g. ‘I was scared that my family could break apart at any time’ or ‘[My caregivers] or other family members were unpredictable’). Three additional items assess minimising responses on a seventh scale (e.g. ‘I had the best family in the world’). Participants are asked about the time of their upbringing and to rate each of the 31 items (e.g. ‘I had enough to eat’) on a 5-point Likert scale (ranging from 1 = never to 5 = very often). The German version of the abuse and neglect scales have been validated.^39^ We found good internal consistency for the total score (ω = 0.95), emotional abuse (ω = 0.89), emotional neglect (ω = 0.92), physical abuse (ω = 0.88), sexual abuse (ω = 0.92), inconsistency experiences (ω = 0.85) and minimisation (ω = 0.80) but low internal consistency for physical neglect (ω = 0.65), which is in line with previous findings.^39,41^ Since the family inconsistency subscale is not frequently used, we examined the proposed seven-factor structure using confirmatory factor analysis, resulting in acceptable fit (comparative fit index CFI = 0.98, Tucker–Lewis index TLI = 0.98, root mean square error of approximation RMSEA = 0.05, standardised root mean squared residual SRMR = 0.08) with all factor loadings ≥0.40 on the intended factors except for the physical neglect item ‘Someone brought me to a physician when I needed it’. We provide prevalence rates for individuals reporting at least moderate levels of childhood abuse and neglect, using the cut-offs initially proposed by Bernstein et al,^38^ which have been widely adopted.^42^

### Statistical analyses

All analyses were conducted in R (R Core Team, 2023, version 4.3.3 for Windows). Average levels of the reported CTQ values were compared between therapy types using Student's *t*-tests or Welch's *t*-tests, as appropriate. Proportions of at least moderate abuse and neglect experiences were compared using χ^2^-tests or Fisher's exact tests, as appropriate. Our hypotheses on the effect of the interaction between therapy type and childhood trauma on changes in depressive symptoms were tested using a linear mixed-effects model approach to account for repeated measurements of each participant. The model included a participant-specific Gaussian random intercept. We accounted for initial depression severity and varying treatment doses by including the sample mean-centred baseline depression (BDI-II) score and total therapy dose (session number) as fixed covariates. Therapy type, childhood trauma, time and their respective interactions were incorporated as fixed effects. A three-way interaction between therapy type, childhood trauma and time was included to evaluate our central hypothesis. Time was assessed as years after treatment start. Therapy type was coded as a factor variable (0 = CBT; 1 = PAT). The modelling approach uses all available data (i.e. not only completers) and is valid under the missing at random (MAR) assumption. Following recommendations,^43^ we report the unstandardised regression coefficients and report 95% confidence intervals alongside *P*-values. We conducted graphical *post hoc* analyses of significant interactions to interpret the slopes.

#### Missing data and sensitivity analyses

For the intention to treat (ITT) sample based on 252 participants, *n* = 6 CTQ assessments (2.38%) were missing at baseline and were handled using listwise deletion. BDI-II data were complete at baseline, but a substantial proportion of data were lost to follow-up: BDI-II data were missing for *n* = 67 participants (26.58%) in year 1, *n* = 103 (40.87%) in year 2, *n* = 103 (40.87%) in year 3, *n* = 132 (52.38%) in year 3 and *n* = 119 (47.22%) in year 5. The percentages reported here are given with reference to the ITT sample. The exact amount of available data for each therapy group at each assessment point is presented in Supplementary Table 1, available at https://doi.org/10.1192/bjp.2024.112. Given the large proportion of missing data, we based our analysis sample on all participants with valid CTQ baseline data and at least two valid BDI-II assessments. This procedure resulted in an analysis sample of *n* = 210 participants. In our main analysis, we assumed the data to be missing at random. In addition, we performed a first sensitivity analysis by repeating all analysis steps based on last observation carried forward (LOCF) imputation. The described procedure mimics the management of missing data for our primary outcome analysis. To increase statistical power, we merged the randomised and preference cells, as no difference in outcomes between the randomised and preference cells was found in our main outcome publications.^25,28^ As reported in Beutel et al,^28^ 1 CBT participant and 17 PAT participants were in ongoing treatment at the 5-year assessment. In a second sensitivity analysis, we excluded all participants still receiving treatment after year 4, so that the last assessment point is an actual follow-up.

## Results

### Trial flow and sample description

In total, 554 people participated in diagnostic interviews, of whom 252 constitute the intention to treat (ITT) sample. We refer to the main outcome publications for a detailed trial flowchart and information about the 302 participants who did not enter the trial.^25,28^ Table 1 shows the baseline information of the ITT sample, stratified by the two treatment conditions. Participants in the CBT and PAT groups did not differ significantly regarding sociodemographic information or childhood trauma reports. At least moderate levels of emotional neglect were most frequently reported (*n* = 135, 53.6%), followed by emotional abuse (*n* = 107, 42.5%). About one-third of participants reported having experienced physical neglect (*n* = 80, 31.7%), and physical abuse experiences were reported by 15.5% of participants (*n* = 39). Sexual abuse was experienced by 24.6% of participants (*n* = 62). Pearson correlation coefficients between the CTQ total and CTQ subscales are reported in Supplementary Table 2. The median session number was 242 for PAT and 59 for CBT. Seventeen participants in PAT and one in CBT were in ongoing treatment at the 5-year outcome assessment.

**Table 1:** Baseline characteristics of the intention to treat (ITT) sample (*N* = 252) separately for cognitive–behavioural therapy (CBT) and psychoanalytic psychotherapy (PAT)^a^

	CBT	PAT	*P*
	*n* = 104	*n* = 148	(*T* or χ^2^)
Age, years: mean (s.d.)	40.11 (10.10)	40.98 (11.10)	0.524
Gender, *n* (%)			
Male	33 (31.7)	49 (33.1)	0.926
Female	71 (68.3)	99 (66.9)	
Main diagnosis, *n* (%)			
Double depression^b^	31 (29.8)	43 (29.1)	0.651
Dysthymia	15 (14.4)	16 (10.8)	
Major depression	58 (55.8)	89 (60.1)	
Comorbid PTSD, *n* (%)	1 (1.0)	3 (2.0)	0.877
Allocation type, *n* (%)			
Preferred	63 (60.6)	101 (68.2)	0.262
Randomised	41 (39.4	47 (31.8)	
German nationality, *n* (%)	89 (85.6)	131 (88.5)	0.619
Job status, *n* (%)			
Full-/part-time	67 (64.4)	101 (68.2)	0.807
Not working	18 (17.3)	19 (12.8)	
In school/training	4 (3.8)	8 (5.4)	
Unemployed	10 (9.6)	15 (10.1)	
Educational level, *n* (%)			
Lower secondary/middle school	33 (31.7)	43 (29.1)	0.577
High school	65 (62.5)	101 (68.2)	
Did not graduate/other	2 (1.9)	1 (0.7)	
Marital status, *n* (%)			
Single	59 (56.7)	87 (58.8)	0.170
Married	22 (21.2)	42 (28.4)	
Separated	17 (16.3)	17 (11.5)	
Widowed	1 (1.0)	0 (0.0)	
Inability to work, weeks during past year: mean (s.d.)	9.63 (14.35)	6.56 (10.51)	0.059
Previous out-patient treatment, *n* (%)			
None	31 (29.8)	36 (24.3)	0.445
1	27 (26.0)	36 (24.3)	
2 or more	41 (39.4)	72 (48.6)	
CTQ total, mean (s.d.)	56.35 (17.93)	58.55 (18.00)	0.345
CTQ emotional abuse, mean (s.d.)	11.87 (5.09)	12.70 (5.37)	0.223
CTQ emotional neglect, mean (s.d.)	14.65 (5.00)	14.97 (5.20)	0.631
CTQ physical abuse, mean (s.d.)	6.82 (3.42)	7.01 (3.13)	0.649
CTQ physical neglect, mean (s.d.)	8.55 (3.51)	8.19 (2.89)	0.378
CTQ sexual abuse, mean (s.d.)	6.58 (3.13)	7.44 (3.79)	0.063
CTQ family inconsistency, mean (s.d.)	7.74 (3.58)	8.11 (3.85)	0.449
BDI-II, mean (s.d.)	32.21 (7.44)	32.05 (8.35)	0.872

PTSD, post-traumatic stress disorder; CTQ, Childhood Trauma Questionnaire; BDI-II, Beck Depression Inventory – II.

a. Missing baseline information for job status (*n* = 10 participants), educational level (*n* = 7), marital status *(n* = 7), inability to work (*n* = 15), previous out-patient treatment (*n* = 9), CTQ scales (*n* = 6), all *P* > 0.05 between treatment groups.

b. Comorbid dysthymia and major depressive disorder.

### Change in depressive symptoms

**Fig. 1:**
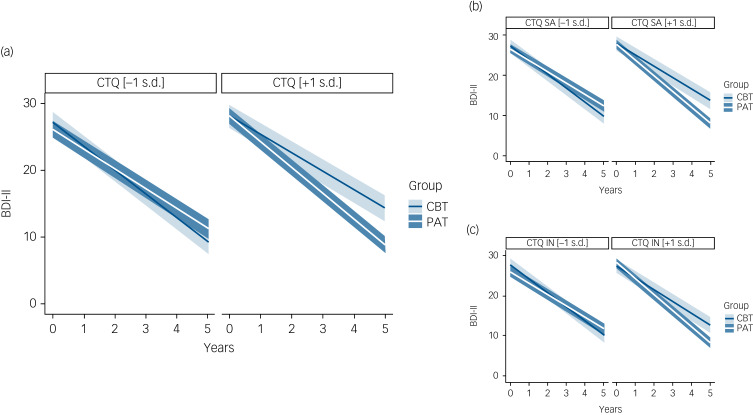
Estimated average (mean; s.e.) decline in depressive symptoms on the Beck Depression Inventory – II (BDI-II) over time, depending on the type of therapy and different levels of childhood trauma measured on the Childhood Trauma Questionnaire (CTQ). Results for the model including (a) the CTQ total, (b) the CTQ subscale sexual abuse (SA) and (c) the CTQ subscale family inconsistency (IN). CBT, cognitive–behavioural therapy; PAT, psychoanalytic psychotherapy.

The results of the multilevel models for the CTQ total scale and its subscales are presented in Table 2. Across all models, there was a main effect of baseline symptom severity, indicating that higher symptom severity at baseline predicted higher symptom severity throughout treatment. We found a statistically significant main effect for a decrease in depressive symptoms over time. In two models a significant two-way interaction between time and treatment type was observed. However, the *post hoc* exploration of the linear decrease in symptoms over time revealed no statistically significant difference between treatment groups (Supplementary Fig. 1), paralleling the results of our main outcome analysis. We therefore conclude that CBT and PAT lead to similar symptom reductions over time, controlling for CTQ (i.e. childhood trauma) levels. There was no statistically significant main effect of the CTQ (total scale and subscales) or its two-way interaction with time. Hence, we did not find evidence indicating that CTQ levels would generally affect symptom courses in long-term therapies. The hypothesis regarding a differential benefit of PAT for participants who experienced childhood trauma was evaluated by examining the effect of the three-way interaction between time, treatment type and CTQ levels on changes in depressive symptoms. Here, a statistically significant interaction was found on the total scale as well as for the two subscales sexual abuse and family inconsistency. For the models testing physical abuse (*P* = 0.080) and physical neglect (*P* = 0.053) a trend for this interaction was observed but did not reach statistical significance. Statistically significant effects were examined graphically. Figure 1 shows that a steeper decline in symptoms was found in PAT compared with CBT at higher childhood trauma levels. At lower childhood trauma levels, both treatment groups achieved similar symptom reductions. We therefore conclude that participants reporting higher levels of childhood trauma profit more from PAT than from CBT. Within the PAT treatment condition, the slope of the decrease in symptoms over time was steeper at higher compared with lower childhood trauma levels, indicating that these participants profit especially well from PAT.

**Table 2:** Results of the linear mixed-effects models on the change in depressive symptoms over time

	CTQ total											
	Estimate (s.e.)	95% CI	*T*	*P*								
Intercept	25.10 (3.35)	18.59 to 31.62	7.48	<0.001								
Baseline BDI-II	4.88 (0.52)	3.86 to 5.89	9.30	<0.001								
Treatment dose	0.07 (0.01)	−0.01 to 0.02	0.83	0.407								
Treatment type	−2.21 (4.6)	−11.16 to 6.73	−0.48	0.632								
Time	−4.55 (0.90)	−6.32 to −2.81	−5.08	<0.001								
CTQ	0.03 (0.06)	−0.08 to 0.14	0.46	0.643								
Treatment type × time	2.51 (1.18)	0.20 to 4.83	2.13	0.034								
Treatment type × CTQ	0.03 (0.07)	−0.11 to 0.17	0.42	0.676								
Time × CTQ	0.02 (0.02)	−0.01 to 0.05	1.54	0.125								
Treatment type × time × CTQ	−0.05 (0.02)	−0.09 to −0.01	−2.42	0.016								

	CTQ emotional abuse				CTQ emotional neglect				CTQ physical abuse			
	Estimate (s.e.)	95% CI	*T*	*P*	Estimate (s.e.)	95% CI	*T*	*P*	Estimate (s.e.)	95% CI	*T*	*P*
Intercept	26.57 (2.78)	21.16 to 31.98	9.55	<0.001	25.97 (3.18)	19.79 to 32.15	8.17	<0.001	25.93 (2.29)	21.49 to 30.38	11.35	<0.001
Baseline BDI-II	4.94 (0.53)	3.91 to 5.96	9.35	<0.001	4.90 (0.52)	3.89 to 5.92	9.41	<0.001	4.94 (0.51)	3.94 to 5.94	9.61	<0.001
Treatment dose	0.01 (0.01)	−0.01 to 0.02	0.80	0.423	0.01(0.01)	−0.01 to 0.02	0.82	0.413	0.01 (0.01)	−0.01 to 0.02	0.90	0.370
Treatment type	−1.64 (3.81)	−9.05 to 5.77	−0.43	0.688	−1.93 (4.23)	−10.25 to 6.38	−0.45	0.652	−1.91(3.43)	−8.59 to 4.76	−0.56	0.579
Time	−4.01(0.74)	−5.47 to −2.56	−5.4	<0.001	−4.30 (0.86)	−5.99 to −2.63	−5.01	<0.001	−4.36 (0.63)	−5.60 to −3.13	−6.92	<0.001
CTQ	0.00 (0.23)	−0.44 to 0.45	0.01	0.991	0.04 (0.20)	−0.36 to 0.44	0.20	0.840	0.09 (0.29)	−0.48 to 0.66	0.31	0.760
Treatment type × time	1.05 (0.94)	−0.78 to 2.88	1.12	0.263	1.16 (1.11)	−1.01 to 3.33	1.05	0.296	1.10 (0.85)	−0.56 to 2.76	1.29	0.197
Treatment type × CTQ	0.10 (0.27)	−0.42 to 0.63	0.38	0.703	0.11 (0.26)	−0.39 to 0.60	0.41	0.681	0.21 (0.40)	−0.56 to 0.99	0.53	0.597
Time × CTQ	0.07 (0.06)	−0.52 to 0.19	1.10	0.270	0.07 (0.06)	−0.04 to 0.18	1.29	0.196	0.16 (0.08)	0.00 to 0.33	1.96	0.051
Treatment type × time × CTQ	−0.11 (0.07)	−0.25 to 0.04	−1.46	0.144	−0.10 (0.07)	−0.24 to 0.04	−1.34	0.180	−0.20 (0.11)	−0.42 to 0.03	−1.72	0.085

	CTQ physical neglect				CTQ sexual abuse				CTQ family inconsistency			
	Estimate (s.e.)	95% CI	*T*	*P*	Estimate (s.e.)	95% CI	*T*	*P*	Estimate (s.e.)	95% CI	*T*	*P*
Intercept	23.55 (2.60)	18.05 to 28.60	9.07	<0.001	26.00 (2.32)	21.50 to 30.50	11.23	<0.001	26.84 (2.45)	22.08 to 31.60	10.96	<0.001
Baseline BDI-II	4.89 (0.52)	3.89 to 5.89	9.48	<0.001	4.99 (0.52)	3.99 to 6.00	9.66	<0.001	4.99 (0.53)	3.97 to 6.02	9.44	<0.001
Treatment dose	0.01(0.01)	−0.01 to 0.02	0.91	0.366	0.01 (0.01)	−0.01 to 0.02	0.77	0.441	0.01 (0.01)	−0.01 to 0.02	0.81	0.417
Treatment type	1.58 (3.80)	−5.81 to 8.96	0.42	0.679	−1.02 (3.30)	−7.45 to 5.39	−0.31	0.758	−3.05 (3.41)	−9.69 to 3.57	−0.89	0.372
Time	−3.82 (0.69)	−5.17 to −2.48	−5.55	<0.001	−3.89 (0.63)	−5.13 to −2.66	−6.17	<0.001	−3.82 (0.66)	−5.12 to −2.54	−5.82	<0.001
CTQ	0.35 (0.27)	−0.19 to 0.88	1.26	0.207	0.09 (0.32)	−0.52 to 0.71	0.30	0.766	−0.03 (0.29)	−0.59 to 0.52	−0.12	0.906
Treatment type × time	1.45 (0.95)	−0.40 to 3.31	1.53	0.127	1.60 (0.81)	0.02 to 3.19	1.97	0.049	1.58 (0.83)	−0.03 to 3.21	1.91	0.056
Treatment type × CTQ	−0.23 (0.38)	−0.97 to 0.51	−0.6	0.549	0.09 (0.38)	−0.65 to 0.84	0.24	0.809	0.34 (0.35)	−0.34 to 1.02	0.97	0.335
Time × CTQ	0.07 (0.08)	−0.08 to 0.22	0.94	0.347	0.10 (0.09)	−0.07 to 0.27	1.12	0.264	0.08 (0.09)	−0.08 to 0.23	0.95	0.341
Treatment type × time × CTQ	−0.21 (0.11)	−0.41 to 0.00	−1.94	0.053	−0.26 (0.11)	−0.48 to −0.05	−2.41	0.016	−0.23 (0.10)	−0.42 to −0.04	−2.35	0.019

CTQ, Childhood Trauma Questionnaire; BDI-II, Beck Depression Inventory – II.

a. Model fit information: CTQ total: Akaike information criterion (AIC) = 6946.89, Bayesian information criterion (BIC) = 7004.94; CTQ emotional abuse: AIC = 6942.55, BIC = 7000.59; CTQ emotional neglect AIC = 6941.74; BIC = 6999.79; CTQ physical abuse: AIC = 6933.78; BIC = 6991.83; CTQ physical neglect: AIC = 6932.95; BIC = 6990.99; CTQ sexual abuse: AIC = 6932.62; BIC = 6990.67; CTQ family inconsistency: AIC = 6934.56; BIC = 6992.6, *N* = 210.

### Sensitivity analyses

Sensitivity analyses are displayed in Supplementary Tables 3 and 4. Overall, the results of the LOCF sensitivity analysis in the analysis sample (*n* = 210) parallel the main analysis, with some exceptions regarding models based on the CTQ subscales: the three-way interaction between time, treatment type and the CTQ subscale sexual abuse was no longer statistically significant (*P* = 0.068), while the three-way interaction in the model incorporating physical neglect was now statistically significant (*P* = 0.021) (Supplementary Table 3). The second sensitivity analysis included only participants who ended treatment prior to or at the 4-year assessment (*n* = 177). Again, the results for the model based on the overall CTQ score are completely in line with our main analysis. The three-way interaction between time, treatment type and the CTQ subscale sexual abuse was no longer statistically significant (*P* = 0.064), the three-way interaction in the model incorporating physical abuse (*P* = 0.016) as well as emotional abuse (*P* = 0.049) now emerged as statistically significant (Supplementary Table 4).

## Discussion

As we expected, childhood trauma had a differential effect on symptom change: participants who reported more childhood trauma improved more in PAT than in CBT over a 5-year period. Clearly, PAT and CBT differed regarding therapeutic technique^25^ and the number of sessions. Previous research found that both the number of sessions and the application of psychoanalytic techniques mediated differences in outcomes following psychoanalytic versus cognitive–behavioural treatments, particularly noting that psychoanalytic techniques led to lasting symptom improvement partially driven by the discussion of early memories.^44^ Although we cannot determine the precise mechanisms of the differential response in our study, we surmise that working in line with the PAT study manual, that is, with biographical narratives with close scrutiny of transference manifestations of procedural or embodied patterns, may help patients not only to gain a consistent narrative of their adverse experiences, but also to make corrective experiences counteracting their interpersonal distrust stemming from the traumatic experiences. The reliable and lasting psychoanalytic setting with high session frequency creates a special opportunity to activate old, often not fully conscious memories and observe their influence on present-day construal and emotional experiences with high emotional intensity and vividness that can have pervasive effects on a person's functioning, as Lane et al^45^ posited. This is a prerequisite for understanding that the inadequate behaviour arose in the traumatic situation and has influenced thinking, feeling and acting without being recognised.

### Comparison with previous research

In line with this theoretical argument, our results expand the evidence and strengthen the findings of the differential benefits of psychodynamic therapies for childhood adversity from a previous study.^21^ In that study, participants reporting more adversity (family unhappiness, parental problems) were more flexible in the therapeutic interaction and willing to engage in self-reflection, according to clinician ratings.^46^ These patients’ ratings of the therapeutic alliance were comparatively favourable in psychodynamic therapies.^47^ Previous qualitative research has shown that patients who attributed their symptoms to their life stories were more likely to benefit from psychodynamic compared with solution-focused therapy.^54^ Thus, there might be a match between the focus of psychodynamic therapy linking and reflecting past and current relationship experiences and a patient's ability and willingness to recall and work through childhood trauma and adversity. Future research efforts will need to disentangle whether the experience of childhood trauma, the ability to recall it or the patient's subjective appraisal of their significance contribute to a greater benefit from psychodynamic therapies.

Indeed, memories are unstable and at times details about traumatic experiences may not be consciously available. Previous research has shown that subtle increases in the reporting of childhood trauma, which can be interpreted as a changed recollection in the severity of childhood trauma or a reappraisal of the events, were associated with improvement during psychodynamic in-patient treatment.^41^ To the best of our knowledge, no similar investigations are available for CBT treatments. However, in psychodynamic therapies the strong focus on understanding a patient's current problems in the context of their upbringing^15^ may increase the likelihood of reflecting on the relationship with caregivers. We surmise that within the context of treating depression, the psychodynamic setting is associated with a greater likelihood that childhood trauma comes up and is worked through intensively. Moreover, patients satisfied with psychodynamic treatment describe ‘getting to the root of things’ and ‘working through trauma’ as important to their recovery’.^48^

### Childhood trauma and differential indication

Although findings regarding treatment effectiveness following childhood trauma are not entirely consistent,^1–3^ childhood trauma has been identified as a negative prognostic indicator in treating depression. Identifying patients with a specific need for a psychoanalytic treatment modality is therefore a major step towards treatment personalisation. Future research needs to replicate our findings using fully randomised designs where childhood trauma is stratified prospectively between CBT and PAT. However, as comparative long-term trials have been^49^ and likely will remain rare, our results are an important entry point for generating future hypotheses and to enrich discussions. As different types of childhood trauma often co-occur, we formulated our hypothesis based on the overall presentation of childhood trauma rather than a specific subtype. This is supported by the CTQ total score proving to be a consistent moderator of treatment effects among our main and sensitivity analyses. Experiences of family inconsistencies emerged as one of the drivers of differential treatment responses. This might indicate that for individuals with these kinds of unsettling recollections of their childhood, there is something specifically beneficial in the way in which psychoanalytic treatment orientations aim to rebuild a life narrative as well as a sense of self in relation to others. Indeed, ongoing experiences of inconsistency while growing up can be understood as a chronic threat that the affected child is unable to truly grasp and understand. This subjective experience constitutes the antithesis of basic trust.^13,14^

### Strengths and limitations

A unique strength of the present study pertains to the analysis of the research question within the different theoretical orientations in one psychotherapy trial with the same measurement points, instruments and other potential sources of variance relating to study design and procedure. The ecological validity of the results can be assumed to be high, as not only were participants able to choose their preferred treatment, but study psychotherapies were comparable to routine care in the sense that the number or frequency of sessions and medication were handled flexibly. Still, the results must be interpreted in light of the study's limitations. The most important limitation pertains to the *post hoc* investigation of our hypothesis: the trial focused on chronic depression, not chronic depression related to childhood trauma. Although baseline levels of childhood trauma were comparable between treatment groups, it was not stratified between the conditions during a randomisation process, limiting the quality of evidence. As the choice of session frequency and treatment duration was made in each therapeutic dyad, we cannot specify on optimal dosage. We entered total dose as a control variable, showing no statistically significant effect. This finding may be read in line with the good-enough level literature, suggesting that treatment length is determined by individual change rates.^50,51^ Yet, such an effect may be obscured by the different standard therapy doses between treatments in our study. The fact that a small number of treatments were ongoing after 5 years was handled by additional sensitivity analyses, which confirmed our findings. Although we were able to reliably distinguish between the two therapeutic interventions,^52^ we could not relate a process assessment of the intervention technique with the outcome. These measures were only available for a subsample (*n* = 137), which did not allow us to distinguish the effects of dose and technique. More research is necessary to delineate the exact techniques through which PAT achieved its effectiveness in participants who had experienced childhood trauma. Operative effect size computation in mixed-effects models and their interpretation is still highly debated^53^ and was therefore not included. Lastly, childhood trauma was assessed via self-report, which can differ from third-party sources of information. In the context of this investigation, one could argue that the subjective experience and its recall rather than historical truths matter. In any case, recollections of adverse childhood experiences can be deemed reliable.^54^

## Supporting information

Krakau et al. supplementary material Krakau et al. supplementary material

## Data Availability

The data supporting the findings of this study are available on reasonable request from the corresponding author. Data are not publicly available as open access data has not been approved in the informed consent and the data contain information that could compromise the privacy of research participants. Analytic code is available from the corresponding author on reasonable request. Research materials are available from the corresponding author on reasonable request.
