# Supplementary material for: Childhood trauma and differential response to long-term psychoanalytic versus cognitive–behavioural therapy for chronic depression in adults
Source: Br J Psychiatry. 2024 Oct;225(4):446–53. doi: 10.1192/bjp.2024.112 (PMC11557288; doi:10.1192/bjp.2024.112)
Supplement: Krakau et al. supplementary material [file S0007125024001120sup001.docx]

**SUPPLEMENT**

| **Table S1. Available outcome data** | | | | | | | | |
| --- | --- | --- | --- | --- | --- | --- | --- | --- |
|  | ITT Sample (N = 252) | | | | Analyzed Sample (N = 210) | | | |
|  | CBT | | PAT | | CBT | | PAT | |
|  | N | M (SD) | N | M (SD) | N | M (SD) | N | M (SD) |
| Baseline | 104 | 32.21 (7.44) | 148 | 32.05 (8.35) | 81 | 31.79 (7.02) | 129 | 31.95 (8.47) |
| Year 1 | 71 | 18.21 (12.06) | 114 | 20.68 (12.51) | 69 | 18.71 (11.86) | 113 | 20.7 (12.56) |
| Year 2 | 58 | 17.48 (12.66) | 91 | 17.51 (11.92) | 56 | 17.25 (12.31) | 89 | 17.29 (11.96) |
| Year 3 | 58 | 14.28 (10.01) | 91 | 15.47 (11.61) | 56 | 14.54 (10.07) | 89 | 15.55 (11.69) |
| Year 4 | 49 | 14.1 (11.47) | 71 | 15.63 (12.16) | 48 | 14.31 (11.48) | 70 | 15.47 (12.17) |
| Year 5 | 50 | 14.22 (11.77) | 83 | 12.73 (12.19) | 49 | 14.43 (11.8) | 83 | 12.73 (12.19) |

| **Table S2. Correlation matrix between the CTQ scales at baseline** | | | | | | |
| --- | --- | --- | --- | --- | --- | --- |
|  | CTQ  total | CTQ  emotional abuse | CTQ  emotional neglect | CTQ  physical abuse | CTQ  physical neglect | CTQ  sexual abuse |
| CTQ emotional abuse | 0.87*** |  |  |  |  |  |
| CTQ emotional neglect | 0.79*** | 0.65*** |  |  |  |  |
| CTQ physical abuse | 0.67*** | 0.51*** | 0.37*** |  |  |  |
| CTQ physical neglect | 0.73*** | 0.49*** | 0.58*** | 0.46*** |  |  |
| CTQ sexual abuse | 0.56*** | 0.38*** | 0.18** | 0.40*** | 0.32*** |  |
| CTQ family inconsistencies | 0.79*** | 0.66*** | 0.59*** | 0.38*** | 0.51*** | 0.35*** |
| *Note. N = 246, *** = p < 0.001, ** = p < 0.01, * = p < 0.05.* | | | | | | |

| **Table S3. Results of the linear mixed effect models on the change in depressive symptoms over time using last observation carried forward imputation** | | | | | | | | | | | | |
| --- | --- | --- | --- | --- | --- | --- | --- | --- | --- | --- | --- | --- |
|  | CTQ total | | | |  |  |  |  |  |  |  |  |
|  | Estimate *(SE)* | 95%-*CI* | *T* | *p* |  |  |  |  |  |  |  |  |
| Intercept | 26.26 (3.57) | 19.33; 33.19 | 7.36 | <0.001 |  |  |  |  |  |  |  |  |
| Baseline BDI-II | 4.88 (0.59) | 3.74; 6.02 | 8.30 | <0.001 |  |  |  |  |  |  |  |  |
| Treatment dose | -0.00 (0.01) | -0.02; 0.02 | -0.06 | 0.953 |  |  |  |  |  |  |  |  |
| Treatment type | -1.23 (4.91) | -10.78; 8.31 | -0.25 | 0.802 |  |  |  |  |  |  |  |  |
| Time | -3.40 (0.70) | -5.06; -2.33 | -5.29 | <0.001 |  |  |  |  |  |  |  |  |
| CTQ | 0.01 (0.06) | -0.11; 0.13 | 0.19 | 0.849 |  |  |  |  |  |  |  |  |
| Treatment type*time | 2.22 (0.91) | 0.44; 4.01 | 2.44 | 0.015 |  |  |  |  |  |  |  |  |
| Treatment type*CTQ | 0.04 (0.08) | -0.11; 0.19 | 0.49 | 0.625 |  |  |  |  |  |  |  |  |
| Time*CTQ | 0.02 (0.01) | -0.01; 0.04 | 1.32 | 0.187 |  |  |  |  |  |  |  |  |
| Treatment type*time*CTQ | -0.04 (0.02) | -0.07; -0.01 | -2.67 | 0.008 |  |  |  |  |  |  |  |  |
|  | CTQ emotional abuse | | | | CTQ emotional neglect | | | | CTQ physical abuse | | | |
|  | Estimate *(SE)* | 95%-*CI* | *T* | *p* | Estimate *(SE)* | 95%-*CI* | *T* | *p* | Estimate *(SE)* | 95%-*CI* | *T* | *p* |
| Intercept | 27.37 (2.93) | 21.67; 33.07 | 9.33 | <0.001 | 27.68 (3.35) | 21.18; 34.19 | 8.27 | <0.001 | 26.21 (2.47) | 21.42; 31.00 | 10.63 | <0.001 |
| Baseline BDI-II | 4.91 (0.59) | 3.77; 6.05 | 8.34 | <0.001 | 4.92 (0.58) | 3.79; 6.06 | 8.43 | <0.001 | 4.88 (0.58) | 3.76; 6.01 | 8.46 | <0.001 |
| Treatment dose | -0.00 (0.01) | -0.02; 0.02 | -0.06 | 0.951 | -0.00 (0.01) | -0.02; 0.02 | -0.04 | 0.967 | 0.00 (0.01) | -0.02; 0.02 | 0.01 | 0.991 |
| Treatment type | -0.59 (4.06) | -8.47; 7.28 | -0.15 | 0.884 | -2.03 (4.54) | -10.85; 6.80 | -0.45 | 0.656 | -0.79 (3.70) | -7.99; 6.39 | -0.22 | 0.829 |
| Time | -3.48 (0.57) | -4.60; -2.36 | -6.09 | <0.001 | -3.47 (0.66) | -4.76; -2.18 | -5.27 | <0.001 | -3.42 (0.48) | -4.36; -2.48 | -7.10 | <0.001 |
| CTQ-Total | -0.04 (0.24) | -0.51; 0.43 | -0.18 | 0.861 | -0.05 (0.22) | -0.47; 0.36 | -0.25 | 0.800 | 0.10 (0.32) | -0.52; 0.72 | 0.30 | 0.762 |
| Treatment type*time | 1.34 (0.72) | -0.28; 2.55 | 1.58 | 0.115 | 1.29 (0.84) | -0.36; 2.93 | 1.53 | 0.127 | 0.92 (0.65) | -0.35; 2.18 | 1.42 | 0.157 |
| Treatment type* CTQ | 0.13 (0.29) | -0.43; 0.69 | 0.46 | 0.645 | 0.20 (0.27) | -0.32; 0.73 | 0.74 | 0.457 | 0.24 (0.43) | -0.59; 1.08 | 0.57 | 0.571 |
| Time*CTQ | 0.06 (0.05) | -0.33; 0.15 | 1.25 | 0.210 | 0.05 (0.04) | -0.04; 0.13 | 1.05 | 0.294 | 0.09 (0.06) | -0.04; 0.21 | 1.39 | 0.165 |
| Treatment type*time*CTQ | -0.11 (0.06) | -0.22; 0.01 | -1.87 | 0.062 | -0.10 (0.05) | -0.20; 0.01 | -1.77 | 0.078 | -0.15 (0.09) | -0.32; 0.01 | -1.79 | 0.074 |
|  | CTQ physical neglect | | | | CTQ sexual abuse | | | | CTQ family inconsistencies | | | |
|  | Estimate *(SE)* | 95%-*CI* | *T* | *p* | Estimate *(SE)* | 95%-*CI* | *T* | *p* | Estimate *(SE)* | 95%-*CI* | *T* | *p* |
| Intercept | 24.77 (2.77) | 19.39; 30.16 | 8.94 | <0.001 | 25.70 (2.49) | 20.87; 30.53 | 10.33 | <0.001 | 27.57 (2.59) |  |  | <0.001 |
| Baseline BDI-II | 4.84 (0.58) | 3.72; 5.96 | 8.38 | <0.001 | 4.94 (0.58) | 3.81; 6.06 | 8.54 | <0.001 | 4.99 (0.59) |  |  | <0.001 |
| Treatment dose | -0.00 (0.01) | -0.02; 0.02 | -0.05 | 0.957 | -0.00 (0.01) | -0.02; 0.02 | -0.08 | 0.938 | -0.00 (0.01) |  |  | 0.951 |
| Treatment type | 2.60 (4.08) | -5.33; 10.53 | 0.64 | 0.525 | 1.40 (3.57) | -5.53; 8.32 | 0.39 | 0.695 | -1.75 (3.65) |  |  | 0.632 |
| Time | -3.40 (0.54) | -4.46; -2.34 | -6.26 | <0.001 | -3.01 (0.49) | -3.96; -2.06 | -6.19 | <0.001 | -3.07 (0.50) |  |  | <0.001 |
| CTQ-Total | 0.25 (0.29) | -0.32; 0.82 | 0.85 | 0.397 | 0.19 (0.34) | -0.47; 0.85 | 0.55 | 0.581 | -0.09 (0.30) |  |  | 0.770 |
| Treatment type*time | 1.46 (0.74) | 0.00; 2.91 | 1.96 | 0.050 | 0.96 (0.62) | -0.25; 2.18 | 1.55 | 0.122 | 1.19 (0.64) |  |  | 0.062 |
| Treatment type* CTQ | -0.18 (0.41) | -0.98; 0.61 | -0.45 | 0.655 | -0.07 (0.41) | -0.87; 0.73 | -0.18 | 0.859 | 0.35 (0.37) |  |  | 0.350 |
| Time*CTQ | 0.07 (0.06) | -0.05; 0.19 | 1.17 | 0.244 | 0.03 (0.07) | -0.10; 0.16 | 0.44 | 0.660 | 0.03 (0.06) |  |  | 0.580 |
| Treatment type*time*CTQ | -0.19 (0.08) | -0.35; -0.03 | -2.31 | 0.021 | -0.15 (0.08) | -0.31; 0.01 | -1.83 | 0.068 | -0.17 (0.07) |  |  | 0.026 |
| Note. *SE* = Standard Error; *CI* = Confidence Interval; Model fit information: CTQ total: AIC = 9250.48, BIC = 9312.14; CTQ emotional abuse: AIC = 9245.8, BIC = 9307;46;  CTQ emotional neglect: AIC = 9246.64; BIC = 9308.30; CTQ physical abuse: AIC = 9241.36; BIC = 9303.02; CTQ physical neglect: AIC = 9237.29; BIC = 9298.96; CTQ sexual abuse: AIC = 9238.38; BIC = 9300.05; CTQ inconsistency experience: AIC = 9238.12; BIC = 9299.78; *N =* 210. | | | | | | | | | | | | |

| **Table S4. Results of the linear mixed effect models on the change in depressive symptoms over time using last observation carried forward imputation** | | | | | | | | | | | | |
| --- | --- | --- | --- | --- | --- | --- | --- | --- | --- | --- | --- | --- |
|  | CTQ total | | | |  |  |  |  |  |  |  |  |
|  | Estimate *(SE)* | 95%-*CI* | *T* | *p* |  |  |  |  |  |  |  |  |
| Intercept | 24.64 (3.35) | 18.13; 31.14 | 7.35 | <0.001 |  |  |  |  |  |  |  |  |
| Baseline BDI-II | 4.91 (0.56) | 3.83; 5.98 | 8.84 | <0.001 |  |  |  |  |  |  |  |  |
| Treatment dose | 0.01 (0.01) | -0.01; 0.03 | 1.35 | 0.179 |  |  |  |  |  |  |  |  |
| Treatment type | -4.47 (4.89) | -13.97; 4.99 | -0.92 | 0.361 |  |  |  |  |  |  |  |  |
| Time | -4.60 (0.91) | -6.39; -2.82 | -5.05 | <0.001 |  |  |  |  |  |  |  |  |
| CTQ | 0.03 (0.06) | -0.08; 0.14 | 0.50 | 0.621 |  |  |  |  |  |  |  |  |
| Treatment type*time | 2.82 (1.29) | 0.29; 5.35 | 2.18 | 0.030 |  |  |  |  |  |  |  |  |
| Treatment type*CTQ | 0.06 (0.08) | -0.09; 0.21 | 0.74 | 0.463 |  |  |  |  |  |  |  |  |
| Time*CTQ | 0.02 (0.02) | -0.01; 0.06 | 1.56 | 0.120 |  |  |  |  |  |  |  |  |
| Treatment type*time*CTQ | -0.06 (0.02) | -0.10; -0.01 | -2.58 | 0.010 |  |  |  |  |  |  |  |  |
|  | CTQ emotional abuse | | | | CTQ emotional neglect | | | | CTQ physical abuse | | | |
|  | Estimate *(SE)* | 95%-*CI* | *T* | *p* | Estimate *(SE)* | 95%-*CI* | *T* | *p* | Estimate *(SE)* | 95%-*CI* | *T* | *p* |
| Intercept | 26.14 (2.80) | 20.71; 31.56 | 9.34 | <0.001 | 25.62 (3.21) | 19.40; 31.84 | 7.99 | <0.001 | 25.60 (2.30) | 21.13; 30.06 | 11.12 | <0.001 |
| Baseline BDI-II | 4.99 (0.56) | 3.90; 6.08 | 8.86 | <0.001 | 4.99 (0.56) | 3.91; 6.06 | 8.98 | <0.001 | 4.99 (0.55) | 3.93; 6.05 | 9.12 | <0.001 |
| Treatment dose | 0.01 (0.01) | -0.01; 0.03 | 1.31 | 0.192 | 0.01 (0.01) | -0.01; 0.03 | 1.27 | 0.205 | 0.01 (0.01) | -0.01; 0.03 | 1.38 | 0.171 |
| Treatment type | -4.00 (4.07) | -11.91; 3.89 | -0.98 | 0.327 | -3.22 (4.51) | -11.98; 5.52 | -0.71 | 0.475 | -3.02 (3.70) | -10.21; 4.16 | -0.82 | 0.416 |
| Time | -4.03 (0.76) | -5.51; -2.55 | -5.32 | <0.001 | -4.33 (0.88) | -6.07; -2.62 | -4.94 | <0.001 | -4.41 (0.64) | -5.66; -3.15 | -6.87 | <0.001 |
| CTQ-Total | 0.01 (0.23) | -0.43; 0.49 | 0.04 | 0.970 | 0.04 (0.21) | -0.35; 0.44 | 0.21 | 0.837 | 0.09 (0.29) | -0.48; 0.65 | 0.30 | 0.765 |
| Treatment type*time | 1.52 (1.02) | -0.48; 3.51 | 1.49 | 0.138 | 1.82 (1.22) | -0.57; 4.21 | 1.49 | 0.137 | 1.67 (0.93) | -0.15; 3.49 | 1.80 | 0.073 |
| Treatment type*CTQ | 0.23 (0.29) | -0.33; 0.79 | 0.80 | 0.425 | 0.14 (0.27) | -0.38; 0.67 | 0.53 | 0.598 | 0.25 (0.42) | -0.56; 1.07 | 0.61 | 0.544 |
| Time*CTQ | 0.07 (0.06) | -0.05; 0.19 | 1.10 | 0.272 | 0.07 (0.06) | -0.04; 0.19 | 1.30 | 0.194 | 0.17 (0.08) | 0.00; 0.33 | 1.99 | 0.047 |
| Treatment type*time*CTQ | -0.16 (0.08) | -0.32; -0.01 | -1.97 | 0.049 | -0.15 (0.08) | -0.30; 0.00 | -1.90 | 0.059 | -0.30 (0.12) | -0.54; -0.06 | -2.41 | 0.016 |
|  | CTQ physical neglect | | | | CTQ sexual abuse | | | | CTQ family inconsistencies | | | |
|  | Estimate *(SE)* | 95%-*CI* | *T* | *p* | Estimate *(SE)* | 95%-*CI* | *T* | *p* | Estimate *(SE)* | 95%-*CI* | *T* | *p* |
| Intercept | 23.07 (2.63) | 17.96; 28.17 | 8.76 | <0.001 | 25.56 (2.31) | 21.08; 30.05 | 11.06 | <0.001 | 26.36 (2.45) | 21.60; 31.12 | 10.74 | <0.001 |
| Baseline BDI-II | 4.88 (0.55) | 3.82; 5.94 | 8.92 | <0.001 | 5.03 (0.55) | 3.97; 6.09 | 9.18 | <0.001 | 5.00 (0.56) | 3.92; 6.09 | 8.97 | <0.001 |
| Treatment dose | 0.01 (0.01) | -0.01; 0.03 | 1.35 | 0.178 | 0.01 (0.01) | -0.01; 0.03 | 1.37 | 0.173 | 0.01 (0.01) | -0.01; 0.03 | 1.34 | 0.181 |
| Treatment type | 1.53 (4.15) | -6.53; 9.58 | 0.37 | 0.713 | -3.82 (3.59) | -10.81; 3.14 | -1.06 | 0.289 | -4.22 (3.60) | -11.21; 2.74 | -1.17 | 0.241 |
| Time | -3.86 (0.71) | -5.25; -2.47 | -5.42 | <0.001 | -3.89 (0.64) | -5.14; -2.65 | -6.12 | <0.001 | -3.89 (0.67) | -5.19; -2.58 | -5.81 | <0.001 |
| CTQ-Total | 0.36 (0.27) | -0.17; 0.89 | 1.31 | 0.191 | 0.10 (0.31) | -0.51; 0.71 | 0.32 | 0.752 | -0.02 (0.28) | -0.57; 0.53 | -0.07 | 0.941 |
| Treatment type*time | 1.03 (1.05) | -1.02; 3.10 | 0.98 | 0.327 | 1.45 (0.90) | -0.60; 2.91 | 1.28 | 0.201 | 1.24 (0.89) | -0.49; 2.97 | 1-39 | 0.163 |
| Treatment type*CTQ | -0.31 (0.42) | -1.11; 0.50 | -0.74 | 0.458 | 0.35 (0.41) | -0.46; 1.15 | 0.85 | 0.394 | 0.38 (0.36) | -0.32; 1.09 | 1.06 | 0.292 |
| Time*CTQ | 0.07 (0.08) | -0.08; 0.23 | 0.96 | 0.336 | 0.10 (0.09) | -0.08; 0.28 | 1.11 | 0.268 | 0.08 (0.08) | -0.07; 0.24 | 1.03 | 0.304 |
| Treatment type*time*CTQ | -0.18 (0.12) | -0.41; 0.06 | -1.47 | 0.143 | -0.23 (0.12) | -0.47; 0.01 | -1.86 | 0.064 | -0.21 (0.10) | -0.41; -0.01 | -1.99 | 0.046 |
| Note. *SE* = Standard Error; *CI* = Confidence Interval; Model fit information: CTQ total: AIC = 5678.53, BIC = 5734.17; CTQ emotional abuse: AIC = 5672.86, BIC = 5728.50;  CTQ emotional neglect: AIC = 5673.25; BIC = 5728.88; CTQ physical abuse: AIC = 5665.47; BIC = 5721.10; CTQ physical neglect: AIC = 5667.54; BIC = 5723.18; CTQ sexual abuse: AIC = 5667.54; BIC = 5723.18; CTQ inconsistency experience: AIC = 5670.64; BIC = 5726.27; *N =* 177. | | | | | | | | | | | | |


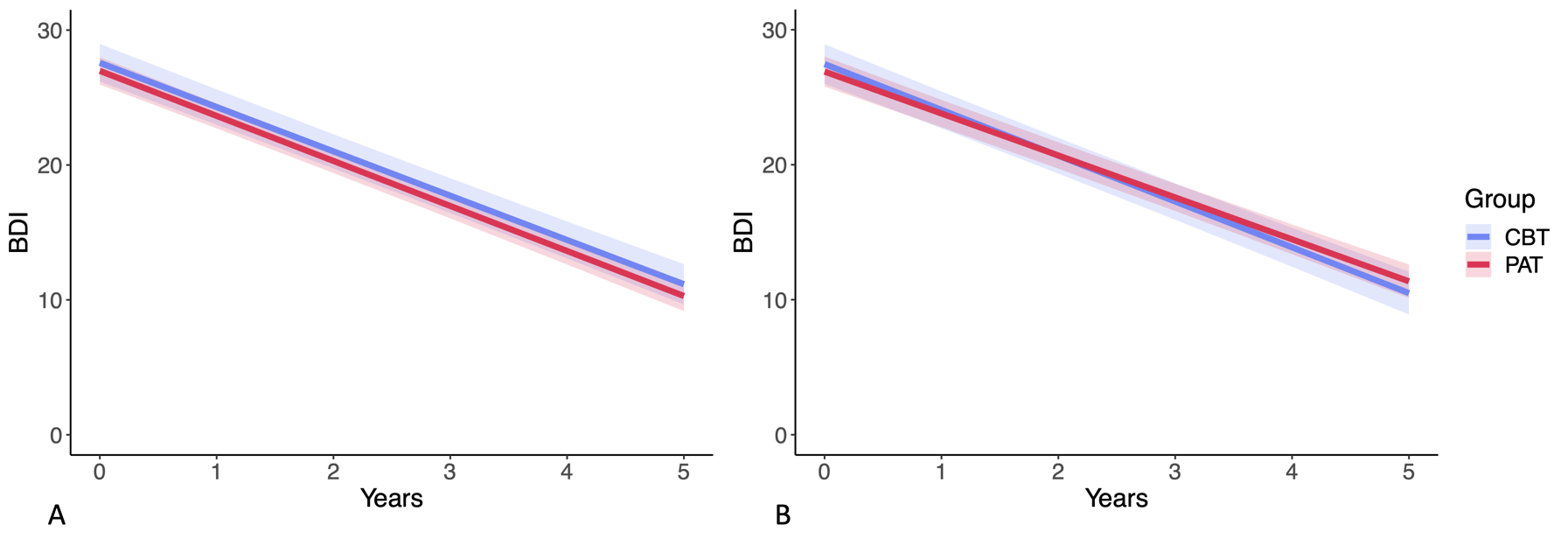


Fig S1. Estimated average (*M, SE*) decline of depressive symptoms (BDI-II) over time, depending on the type of therapy, cognitive behavioral (CBT) and psychoanalytic (PAT) psychotherapy. Panel A shows the results for the model including the CTQ total and Panel B for the CTQ subscale sexual abuse. The post-hoc exploration of the two-way interactions shows a similar decline in symptoms over time in both therapy groups.
